# The relationship between body mass index, binge eating disorder and suicidality

**DOI:** 10.1186/s12888-018-1766-z

**Published:** 2018-06-15

**Authors:** Kristal Lyn Brown, Jessica Gokee LaRose, Briana Mezuk

**Affiliations:** 1 Department of Health Behavior and Policy, Virginia Commonwealth University School of Medicine, Richmond, VA USA; 2 Department of Family Medicine and Population Health, Division of Epidemiology, Virginia Commonwealth University School of Medicine, Richmond, VA USA; 3 Department of Epidemiology, University of Michigan School of Public Health, Ann Arbor, MI USA

**Keywords:** Suicide, Binge eating disorder, Population-based, Gender, Obesity

## Abstract

**Background:**

While restrictive and compensatory eating disorders (e.g. anorexia and bulimia) are associated with elevated risk of suicide, less is known about binge eating disorder (BED). There is suggestive evidence of a U-shaped relationship between body mass index (BMI) and completed suicide, but fewer studies on suicidal ideation or attempts. This study examined the association between BED, BMI, and suicidality, and assessed whether these relationships varied by gender.

**Methods:**

Data come from the Collaborative Psychiatric Epidemiologic Surveys (*N* = 14,497). Binge episodes and BED were assessed using the Composite International Diagnostic Inventory (CIDI). BMI was calculated from self-reported height and weight. Suicidal ideation/attempts were assessed using the CIDI. Weighted logistic regression was used to assess the association between binging/BED, BMI and suicidality. Interaction terms were used to assess whether the relationship between BMI and suicidality was moderated by binging/BED, and whether the relationships between binging/BED and BMI differed by gender.

**Results:**

One-third of adults with BED had a history of suicidality, compared to 19% of those without. Both binging (OR: 1.95, 95% CI: 1.50–2.53) and BED (OR: 2.01, 95% CI: 1.41–2.86) were associated with suicidality in fully-adjusted models. BMI was associated with suicidality in a curvilinear manner, and this relationship was exacerbated by binging/BED (OR_Binge eating x BMI_: 1.04, 95% CI: 1.01–1.09, *p* < 0.05). The relationship between BMI and suicidality did not differ by gender (OR_gender x BMI_: 1.00, *p* < 0.770). However, the relationship between binge eating and suicidality was stronger for women relative to men (OR_gender X binge_: 1.87, *p* < 0.012).

**Conclusions:**

Binge eating, even below the threshold for BED, is associated with suicidality. BMI is associated with suicidality in a curvilinear manner, and the BMI-suicidality relationship is potentiated by binge eating/BED. Findings support the thoughtful integration of psychiatric care into weight loss programs for adults with a history of binging behavior.

**Electronic supplementary material:**

The online version of this article (10.1186/s12888-018-1766-z) contains supplementary material, which is available to authorized users.

## Background

Binge Eating Disorder (BED) is characterized by consuming large amounts of food in a short period of time with a marked loss of control [1]. BED is the most common eating disorder in the United States (lifetime prevalence: 3.5% for women and 2% for men, although some estimates range as high as 8%) [1, 2]. Relatively little is known about the course and correlates of this condition as compared to other eating disorders (i.e., anorexia nervosa, bulimia nervosa) as BED was only included as a distinct diagnostic category in the 2013 edition of the Diagnostic and Statistical Manual of Mental Disorders (DSM) [3].

Eating disorders, including BED, are often co-morbid with other forms of psychopathology. For example, adults with BED report higher levels of anxious and depressive symptomology compared to adults without BED [4]. As with psychopathology in general, eating disorders are associated with elevated risk of suicidal behavior [4–7]. For example, numerous epidemiologic studies have shown that anorexia and bulimia nervosa are associated with risk of both attempted and completed suicide; [4, 6] however, only a handful of studies have examined the relationship between BED and suicidal behaviors [4, 8]. In a study of women drawn from the Swedish registries, Pisetsky and colleagues (2013) reported that the risk of attempted suicide associated with BED was elevated similar to other eating disorders, although this estimate was based on only 64 cases of BED [5]. In their meta-analysis, Preti et al. (2010) reported that BED was not associated with completed suicide, although this was also based on a relatively small number of cases [6]. Finally, a more recent study by Suokas et al. (2013) using Finnish registry data reported that suicide mortality was not elevated among persons treated for BED [7]. To our knowledge, the relationship between BED and suicidality has not been examined in a population-based sample of US adults.

The relationship between obesity and psychopathology is complex and remains poorly understood [9]. Unlike other eating disorders (i.e. bulimia, anorexia), individuals with BED do not engage in restrictive or compensatory behaviors (e.g., excessive exercise, laxatives, vomiting). As a result, individuals with BED are at a higher risk for gaining weight and developing obesity. While most studies indicate that extreme obesity (i.e., BMI ≥ 35 kg/m^2^) is positively associated with depression and other forms of psychopathology, [10–13] several reports suggest that the relationship between BMI and psychopathology is non-linear. For example, most studies report little or no difference in the prevalence of depression for BMI in the overweight and class 1 obesity range (i.e., BMI between 25 kg/m^2^ and 35 kg/m^2^) relative to normal weight, [11, 12, 14] and several reports indicate that overweight is associated with lower likelihood of depression relative to normal or underweight [11, 13, 15, 16] particularly for non-Hispanic white populations [17]. The relationship between depression and obesity also appears to be more pronounced for women relative to men [18].

Consistent with this non-linear relationship between BMI and psychopathology, epidemiologic studies using mortality registries consistently report an inverse or inverted J-shaped relationship between BMI and completed suicide, such that suicide risk is highest among individuals with BMI < 20 kg/m^2^ (and, ≥35 kg/m^2^, in those that report a J-shaped relationship), with lowest risk in the overweight and moderate obesity range [19–24]. A recent meta-analysis of 38 population-based studies reported that underweight was associated with elevated risk of suicide, and that overweight and obesity were associated with significantly lower risk of suicide, relative to normal weight [25]. While a handful of smaller studies indicate that extreme obesity (BMI ≥ 40 kg/m^2^) is positively associated with suicidal behavior, [25, 26] most studies report an inverse linear relationship with BMI and suicidal ideation and attempts [26, 27]. Similar to the relationship between depression and obesity, there is suggestive evidence that this relationship between BMI and suicide may vary by gender [28]. However, few, if any, of these studies of BMI and completed suicide have accounted for history of eating disorders.

Suicide prevention requires the identification of novel risk factors for suicidal behaviors. Few large, population-based studies have examined whether binge eating behavior contributes to the relationship between BMI and suicidality. To this end, and building on prior research, the aim of this study was to determine the association between binge eating/BED, BMI and suicidality (i.e., ideation and attempts). We evaluated three hypotheses: (1) BED is associated with elevated likelihood of suicidality, (2) BMI is associated with likelihood of suicidality in a non-linear manner, and (3) The relationship between BED and suicidality is exacerbated by BMI. We also explored whether these relationships differed by gender.

## Methods

### Participants and procedures

Data are from the 2001–2003 Collaborative Psychiatric Epidemiologic Surveys (CPES). The CPES is a set of three nationally-representative cross-sectional householdsurveys (The National Comorbidity Survey Replication (NCS-R), The National Survey of American Life (NSAL), and the National Latino and Asian American Study (NLAAS)) conducted to estimate the prevalence of psychopathology in the adult (age ≥ 18) population and to assess treatment patterns, with specific attention to racial/ethnic minorities [29–31]. Additional details about the CPES study design and sampling approach are described elsewhere [29–31]. The CPES data used for this analysis are available through the Inter-University Consortium for Political and Social Research: https://www.icpsr.umich.edu/icpsrweb/ICPSR/studies/20240.

This analysis was limited to individuals with complete data on BMI, BED, and suicidality (*N* = 14,497), which represents 72% of the total CPES sample. Those excluded from the analytic sample (*N* = 5516) were older, more likely to be male, more likely to be white, and had more education than those included; poverty-to-income ratio and BMI were similar (Additional file 1: Table S1). Most of those excluded were from the NCS-R, which by design only asked the complete set of BED items on a random subset of 2980 participants [32].

### Measures

#### Exposure ascertainment

Lifetime history of BED (with hierarchy) was assessed using the World Health Organization’s (WHO) expanded version of the Composite International Diagnostic Interview (CIDI) for DSM-IV [2]. BED case status was indicated if respondents endorsed binge eating, defined as (a) recurrent episodes of eating, in a discrete (2-h) period, an amount of food that is definitely larger than most people would eat during a similar period of time accompanied by (b) a sense of lack of control over eating, and (c) three or more cognitive or affective feelings during the binge (i.e., eating rapidly, eating until uncomfortably full, eating when not feeling hungry, eating alone due to feeling embarrassed by the amount of food consumed, feeling disgusted with oneself, depressed, or very guilty after overeating, or marked distress). These binge episodes had to occur at least 2 days a week for 6 months, and the binging could not be associated with use of compensatory behaviors (i.e. purging, fasting, excessive exercise), consistent with DSM-IV criteria. Individuals with bulimia or anorexia nervosa were excluded from the BED hierarchy diagnosis. Lifetime history of any binge episode was defined as criteria a and b only, and with duration of two days/week for at least three months. BMI (kg/m^2^) was calculated from self-reported weight and height. It was treated as a continuous variable (centered on the sample mean: 27 kg/m2). As a sensitivity analysis we also evaluated BMI as a categorical variable using WHO categories (< 18.5, 18.5 to < 25 (reference group), 25.0 to < 30 and ≥ 30).

#### Outcome ascertainment

Lifetime suicidality was indexed by a CIDI module that assessed suicidal ideation / intent (i.e., “seriously thought about committing suicide” or “made a plan for committing suicide”) and suicide attempt, including the age of onset and recency of suicidality. For this analysis two dichotomous variables were created: for the main analysis we examined lifetime (ever/never) suicidality (ideation and/or intent), and for the sensitivity analyses we examined past-year suicidality; for the sensitivity analysis individuals who endorsed suicidality only prior to the past year were excluded (*n* = 2030).

We also conducted a post-hoc analysis examining lifetime history of attempting suicide as an outcome. Because of the skip-pattern of the CIDI, history of suicide attempt was only asked of those who endorsed suicidality; however, for this analysis we created a new variable indexing lifetime history of suicide attempt by recoding respondents who had not seriously considered suicide as a “no” for history of attempting suicide, as prior studies of this outcome have done. Due to some missing data on the attempt question, this analysis was limited to 13,079 respondents.

#### Covariates

All covariates were assessed by self-report and included age (in years, mean-centered at 43.4), race/ethnicity, gender, marital status, education and Income-to-needs ratio. Race/ethnicity was categorized as Asian, Hispanic, Black, and Non-Latino White, with Black as the reference group. Education was categorized as high school education or less (reference group) vs. more than high school. Marital status was categorized as currently married (reference group), formerly married, and never married. Income-to-needs ratio is a measure of socioeconomic status calculated by dividing household income by the Census poverty threshold for that household size, [33] categorized into quintiles. We also considered smoking and medical comorbidities as confounders. Lifetime history of medical conditions (including arthritis, chronic pain, headaches, stroke, heart disease, hypertension, chronic lung disease, diabetes, and cancer) were summed and was categorized as zero (reference group), one, two, and three or more conditions for analysis. Smoking status was available on a subset of participants (*N* = 9648) and was categorized as current, former, and never smoker (reference group).

### Analytic approach

Initial bivariate relationships between BED, suicidality and covariates were assessed using Scott-Rao chi-square tests. To address the first hypothesis, weighted logistic regression models were fit to test the association between binge eating behavior and lifetime history of suicidality as the dependent variable. Three nested models were fit for both BED and binge episodes: Model 1 was unadjusted, Model 2 was adjusted for BMI. Model 3 was adjusted for BMI and sociodemographic characteristics, and Model 4 was additionally adjusted for number of chronic health conditions; we also fit models additionally adjusted for smoking status as a sensitivity analysis. Weighted logistic regression models were fit to assess the relationship between BMI and suicidality using a similar nested model approach. To test the second hypothesis, we evaluated whether a quadratic term on BMI improved model fit, indicating a curvilinear relationship with suicidality. To test the third hypothesis, logistic regression models were fit that included interaction terms between BED (and binge eating) and BMI. To evaluate whether these relationships were similar for men and women these models were then fit within strata of sex. As a sensitivity analysis, all models were refit to examine the relationship between BED, binge eating, and BMI with past-year suicidality. Finally, we conducted two post-hoc sensitivity analyses: first, we refit all models examining binge eating as the exposure while excluding the 271 cases of BED from the analysis; and second, we refit all models to examine the outcome of lifetime history of suicide attempt rather than suicidality.

Goodness-of-fit was assessed using the Wald test. All analyses were conducted using STATA/IC 11.2 survey procedures to account for the complex sampling design and all *p*-values refer to two-tailed tests.

## Results

Approximately 4% of adults had a lifetime history of binge eating and 1.9% had a history of BED (48% of those who had history of bingeing) (Table 1). Respondents with BED were younger, predominantly female and were more likely to have obesity relative to those without BED. Approximately 20% had a lifetime history of suicidality, with 4.0% in the past year, and 7.8% had attempted suicide at some point in their life. Among those with a history of binge eating, one-third (34.2%) reported ever thinking about suicide, nearly one in five (18.6%) had ever attempted suicide, and 10.1% experienced suicidality in the past year. These proportions were similar (37.5, 20.5 and 8.9%, respectively) for BED. BMI was strongly right-skewed, and overall 26% of respondents had a BMI in the obesity range.

**Table 1:** Sample Characteristics by lifetime history of binge episode and BED: Collaborative Psychiatric Epidemiologic Surveys

	Lifetime binge episode	Never binge episode	Lifetime BED	Never BED
N	659 (3.9)	13,838 (96.1)	271 (1.9%)	14,226 (98.1%)
Age (Mean, SE)	38.6 (1.0)	43.0 (0.3)	40.4 (1.4)	42.9 (0.3)
Female gender	424 (62.6)	8104 (54.7)	194 (68.2)	8334 (54.8)
Race				
Asian	82 (6.9)	2071 (6.6)	26 (4.1)	2127 (6.6)
Hispanic	192 (22.0)	2936 (15.9)	79 (19.3)	3049 (16.0)
Black	258 (22.0)	5243 (17.3)	94 (16.7)	5407 (17.5)
Non-Latino White	127 (49.1)	3588 (60.3)	72 (60.0)	3643 (60.0)
Marital status				
Currently married	309 (51.0)	7396 (57.2)	133 (53.1)	7572 (57.1)
Divorced/widowed	148 (20.3)	3040 (19.5)	61 (18.8)	3127 (19.6)
Never married	202 (28.7)	3402 (23.3)	77 (28.1)	3527 (23.4)
Education				
≤ 12 years	380 (53.6)	7011 (50.3)	160 (48.8)	7231 (50.4)
> 12 years	279 (46.5)	6827 (49.7)	111 (51.2)	6995 (49.6)
Income-to-needs ratio				
Quintile 1	248 (29.8)	3893 (23.7)	99 (25.0)	4042 (24.0)
Quintile 2	103 (14.8)	2419 (16.3)	43 (14.5)	2479 (16.2)
Quintile 3	132 (24.0)	3339 (25.3)	53 (26.2)	3418 (25.3)
Quintile 4	75 (12.7)	1750 (15.1)	32 (14.4)	1793 (15.0)
Quintile 5	101 (18.8)	2437 (19.6)	44 (19.9)	2494 (19.6)
Smoking status				
Never	201 (43.1)	4814 (46.6)	94 (42.6)	4921 (46.6)
Former	111 (30.1)	2293 (27.2)	51 (38.3)	2353 (27.1)
Current	111 (26.8)	2118 (26.2)	41 (19.2)	2188 (26.3)
Number of chronic conditions				
None	371 (43.3)	8507 (46.2)	138 (37.7)	8740 (46.3)
One	130 (21.0)	2421 (22.3)	57 (21.6)	2494 (22.3)
Two	63 (12.1)	1559 (16.0)	27 (11.6)	1595 (15.9)
Three or more	95 (23.7)	1351 (15.4)	49 (29.0)	1397 (15.5)
BMI kg/m^2^ (Mean, SE)	29.8 (0.4)	27.1 (0.1)	31.0 (0.6)	27.2 (0.1)
BMI categories				
< 25 kg/m^2^	183 (26.5)	5606 (40.7)	58 (17.0)	5731 (40.6)
25 to < 30 kg/m^2^	195 (33.1)	4704 (33.4)	77 (35.8)	4822 (33.3)
30 to < 35 kg/m^2^	143 (20.2)	2214 (16.2)	71 (26.2)	2286 (16.2)
≥ 35 kg/m^2^	138 (20.2)	1314 (9.6)	65 (20.9)	1387 (9.8)
Lifetime history of suicidality	198 (34.2)	2007 (19.0)	86 (37.5)	2119 (19.3)
Lifetime history of suicide attempt	89 (18.6)	698 (7.4)	39 (20.5)	748 (7.6)
Past-year suicidality	42 (10.1)	341 (3.8)	16 (8.9)	367 (4.0)

Values are unweighted N, weighted row percentages unless otherwise noted. N for smoking = 9648

Concerning the first hypothesis, both binge eating (Crude Odds Ratio [OR]: 2.21, 95% Confidence Interval [CI]: 1.67–2.92, *p* < 0.001; Adjusted OR (AOR): 1.95, 95% CI: 1.50–2.53, *p* < 0.001) and BED (Crude OR: 2.51, 95% CI: 1.79–3.50, *p* < 0.001; AOR: 2.01, 95% CI: 1.41–2.86, *p* < 0.001) were significantly associated with lifetime suicidality (Tables 2 and 3). Adding BMI to these models did not substantially attenuate the relationship between binge eating/BED and suicidality. Findings from the post-hoc sensitivity analysis of excluding cases of BED from the models examining binge eating were consistent with these findings (Additional file 1: Table S2). Results were also similar when examining the outcome of past-year suicidality.

**Table 2:** Relationship between binge eating, BMI, and suicidal behavior

	Model 1 OR (95% CI), *p*-value	Model 2 OR (95% CI), *p*-value	Model 3 OR (95% CI), *p*-value	Model 4 OR (95% CI), *p*-value	Model 5 OR (95% CI), *p*-value
Outcome: Lifetime suicidality					
Lifetime binge episode	2.21 (1.67–2.92), *p* < 0.001	2.11 (1.62–2.74), *p* < 0.001	2.09 (1.61–2.73), *p* < 0.001	1.95 (1.50–2.53), *p* < 0.001	1.80 (1.34–2.42), *p* < 0.001
BMI (mean centered)		1.00 (0.99–1.02), *p* < 0.528	1.00 (1.00–1.03), *p* < 0.015	1.02 (0.99–1.02), *p* < 0.217	1.01 (1.00–1.03), *p* < 0.072
BMI^2^		1.00 (1.00–1.01), *p* < 0.003	1.00 (1.00–1.01), *p* < 0.219	1.00 (0.99–1.00), *p* < 0.218	1.00 (0.99–1.00), *p* < 0.239
N	14,497	14,497	14,497	14,497	9648
Outcome: Lifetime suicide attempt					
Lifetime binge episode	2.84 (1.92–4.20), *p* < 0.001	2.72 (1.84–4.03), *p* < 0.001	2.50 (1.71–3.65), *p* < 0.001	2.28 (1.58–3.29), *p* < 0.001	2.03 (1.32–3.10), *p* < 0.001
BMI (mean centered)		1.00 (0.98–1.02), *p* < 0.744	1.01 (0.99–1.04), *p* < 0.211	1.00 (0.98–1.02), *p* < 0.848	1.01 (0.99–1.04), *p* < 0.318
BMI^2^		1.00 (1.00–1.01), *p* < 0.002	1.00 (1.00–1.01), *p* < 0.161	1.00 (1.00–1.01), *p* < 0.122	1.00 (1.00–1.01), *p* < 0.099
N	13,079	13,079	13,079	13,079	8577
Outcome: Past-year suicidality					
Lifetime binge eating	2.80 (1.72–4.56), *p* < 0.001	2.79 (1.76–4.42), *p* < 0.001	2.51 (1.68–3.76), *p* < 0.001	2.33 (1.53–3.55), *p* < 0.001	2.18 (1.36–3.51), *p* < 0.002
BMI (mean centered)		0.99 (0.96–1.02), *p* < 0.350	1.01 (0.98–1.05), *p* < 0.402	1.01 (0.97–1.04), *p* < 0.865	1.01 (0.97–1.05), *p* < 0.557
BMI^2^		1.00 (1.00–1.01), *p* < 0.001	1.00 (1.00–1.01), *p* < 0.166	1.01 (1.00–1.01), *p* < 0.155	1.00 (1.00–1.01), *p* < 0.153
N	12,675	12,675	12,675	12,675	8267

*OR* Odds ratio, *95% CI* 95% Confidence Interval

Model 1: Unadjusted. Model 2: Adjusted for BMI and BMI^2^. Model 3: Adjusted for BMI, race, gender, age, education, marital status, income to needs ratio. Model 4: Adjusted for BMI, race, gender, age, education, marital status, income to needs ratio and number of chronic conditions. Model 5: Adjusted for BMI, race, gender, age, education, marital status, income to needs ratio, number of chronic conditions and smoking status

**Table 3:** Relationship between BED, BMI, and suicidal behavior

	Model 1 OR (95% CI), *p*-value	Model 2 OR (95% CI), *p*-value	Model 3 OR (95% CI), *p*-value	Model 4 OR (95% CI)
Outcome: Lifetime suicidality				
Lifetime BED	2.51 (1.79–3.50), *p* < 0.001	2.35 (1.72–3.23), *p* < 0.001	2.10 (1.52–2.90), *p* < 0.001	2.01 (1.41–2.86), *p* < 0.001
BMI (mean centered)		1.00 (0.99–1.02), *p* < 0.507	1.01 (0.99–1.02), *p* < 0.212	1.01 (1.00–1.03), *p* < 0.075
BMI^2^		1.01 (1.00–1.01), *p* < 0.003	1.00 (1.00–1.00), *p* < 0.211	1.00 (1.00–1.01), *p* < 0.235
N	14,497	14,497	14,497	9648
Outcome: Lifetime suicide attempt				
Lifetime BED	3.11 I2.11–4.59), *p* < 0.001	2.96 (2.02–4.34), *p* < 0.001	2.40 (1.73–3.32), *p* < 0.001	2.31 (1.60–3.32), *p* < 0.001
BMI (mean centered)		1.00 (0.98–1.02), *p* < 0.801	1.00 (0.98–1.02), *p* < 0.813	1.01 (0.99–1.04), *p* < 0.311
BMI^2^		1.00 (1.00–1.01), *p* < 0.002	1.00 (1.00–1.00), *p* < 0.122	1.00 (1.00–1.01), *p* < 0.098
N	13,079	13,079	13,079	8577
Outcome: Past-year suicidality				
Lifetime BED	2.37 (1.12–5.03), *p* < 0.025	2.36 (1.13 0 4.92), *p* < 0.022	2.05 (1.14–3.67), *p* < 0.017	1.91 (1.04–3.53), *p* < 0.039
BMI (mean centered)		0.99 (0.96–1.02), *p* = 0.430	1.01 (0.97–1.04), *p* = 0.822	1.01 (0.97–1.05), *p* = 0.537
BMI^2^		1.00 (1.00–1.01), *p* < 0.001	1.00 (1.00–1.01), *p* < 0.173	1.00 (1.00–1.01), *p* = 0.168
N	12,675	12,675	12,675	8267

*OR* Odds ratio, *95% CI* 95% Confidence Interval

Model 1: Unadjusted. Model 2: Adjusted for BMI and BMI^2^. Model 3: Adjusted for BMI, race, gender, age, education, marital status, income to needs ratio, and number of chronic conditions. Model 4: Adjusted for BMI, race, gender, age, education, marital status, income to needs ratio, number of chronic conditions, and smoking status

**Fig. 1:**
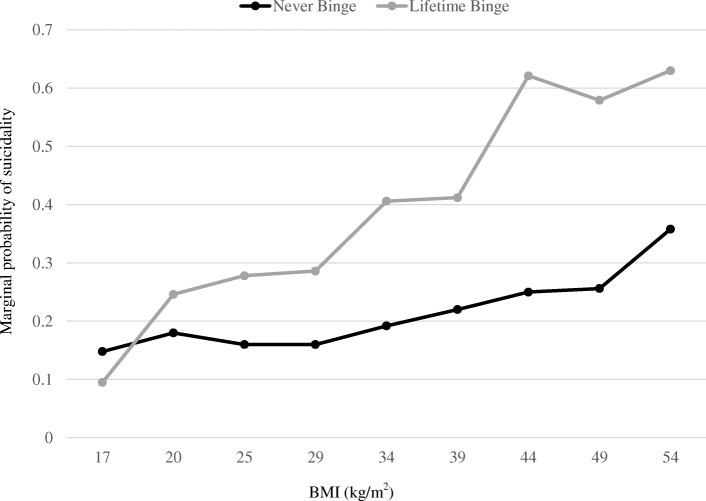
Probability of lifetime suicidality by lifetime binge eating behavior at select BMI. Marginal predicted probability of lifetime suicidal ideation/attempt by lifetime history of binge eating behavior by select BMI. Values are estimated at the sample mean for all model covariates (age, gender, race/ethnicity, marital status, income-to-needs ratio and chronic conditions). *N* = 14,497

The quadratic term on BMI was significant in the crude model (*p* < 0.004), and Fig. 1 illustrates the curvilinear relationship between BMI and lifetime suicidality by binge eating status. There was a modest but statistically significant relationship between BMI and suicidality (Additional file 2: Figure. S1), which was influenced by both number of chronic conditions and smoking status (Additional file 1: Table S3). Modeling BMI as a 4-level categorical variable illustrated a similar non-linear relationship with suicidality (Additional file 1: Table S4). These relationships were consistent when examining suicide attempts and past-year suicidality. Additional file 3: Figure S2 illustrates the curvilinear relationship between BMI and past year suicidality by lifetime binge eating status.

Turning to the third hypothesis, the interaction term between BMI and binge eating on likelihood of suicidality was statistically significant, even in fully-adjusted models (OR_Binge eating x BMI_: 1.04, 95% CI: 1.01, 1.09, *p* < 0.021). The analysis of suicide attempt was similar (OR_Binge eating x BMI_: 1.07, 95% CI: 1.01, 1.14, *p* < 0.018). This interaction is illustrated by Fig. 1, which shows that the relationship between binge eating and suicidality was strongest for those with higher BMI. For example, for adults with a BMI one standard deviation below the sample mean (approximately 20 kg/m^2^) the relative odds of suicidality associated with binge eating was 1.38 (*p* = 0.136); however, for those with a BMI 1.5 standard deviation above the sample mean (approximately 34 kg/m^2^) the relative odds of suicidality associated with binge eating was 2.69 (*p* < 0.001).

**Fig. 2:**
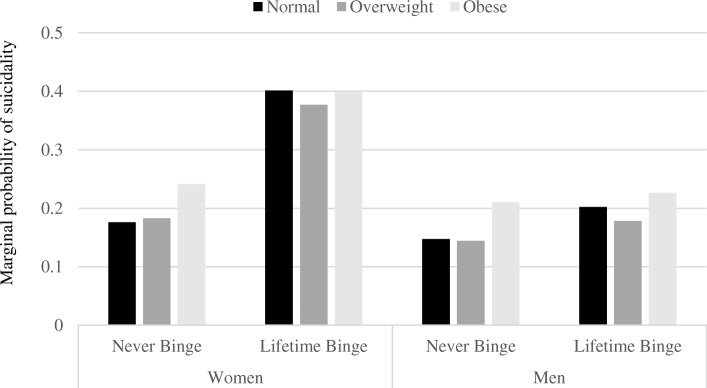
Marginal probability of suicidality by gender and lifetime binge eating behavior. Marginal predicted probability of suicidal ideation/attempts by lifetime history of binge eating behavior by gender and BMI category. Values are estimated at the sample mean for all model covariates (age, gender, race/ethnicity, marital status, income-to-needs ratio and chronic conditions). *N* = 14,497

Finally, we examined whether these relationships were consistent for men and women. There was no evidence that the relationship between BMI and suicidality differed by gender (OR_gender x BMI_: 1.00, *p* < 0.770). However, the relationship between binge eating and suicidality was stronger for women relative to men (OR_gender X binge_: 1.87, *p* < 0.012). Figure 2 illustrates the relationship between binge eating and lifetime suicidality by gender at normal, overweight and obesity levels of BMI from fully-adjusted models. Results from the post-hoc analysis of lifetime suicide attempt were similar (OR_gender X binge_: 2.66, *p* < 0.011).

## Discussion

The primary finding from this study is that both binge eating behaviors and BMI are independently related to suicidal ideation and attempts among US adults. The relationship between BED and suicidality was not attenuated by BMI, indicating that BMI does not substantially explain the association between binge eating and suicidal behavior. However, there was evidence that the relationship between binge eating/BED and suicidality was exacerbated by high BMI. Consistent with prior work on BMI and completed suicide, the relationship between BMI and suicidality was non-linear, with highest risk associated with obesity relative to overweight, but with little difference in suicidality between underweight, normal, and overweight. These relationships were present for both genders, but the association between binge eating and suicidality was stronger for women relative to men. To our knowledge, this is the largest study to examine the relationship between BED, BMI, and suicidality in a diverse, nationally-representative sample of US adults.

The finding that binge eating/BED is associated with suicidality is consistent with the broader literature on eating disorders and associated psychiatric comorbidities [8]. Nearly one-third of women with BED report a lifetime history of suicidal Ideation and 15% had attempted suicide [34]. Several reports have linked binge eating behaviors with mood disorders, [32] novelty-seeking, [35] and impulsiveness, [5, 36] which have in turn been linked to suicidal behaviors. To our knowledge, this study is the first to suggest that the relationship between binge eating and suicidality is moderated by BMI.

The linkages between BMI and suicidality are complex [24]. We found that the relationship between BMI and suicidality did not differ by gender, in contrast to several recent reports. Gao (2013) reported that the association between BMI and suicidality varied by gender and depression status, with an inverse relationship between BMI and suicide attempts among men regardless of depression history; and a curvilinear relationship among women, with higher incidence of attempts among low-BMI relative to normal weight women without history of depression, but a U-shared relationship among women with depression [19]. Kim et al. (2016) also reported that the relationship between BMI and suicidality was curvilinear and varied by gender in a large sample of Korean middle-aged adults [37]. Results from studies of BMI and suicidality among adolescent or young adult samples have generally been smaller and results are mixed [25, 38, 39].

### Strengths and limitations

The cross-sectional nature of our study precludes inferences regarding causality. However, our findings are consistent with prior literature on BED (and eating disorders more broadly) and suicidality, as well as BMI and completed suicide. Our analyses examining past-year suicidality and lifetime history of suicide attempt were consistent with our main results. BMI was calculated from self-reported weight and height, which tends underestimate BMI; [40] individuals with eating disorders tend give a more accurate account of their weight, likely because of greater weight-checking [40]. This analysis did not adjust for comorbid psychiatric conditions (i.e., major depression, general anxiety disorder) because these conditions are likely mediators of the BED (and potentially BMI) – suicidality relationship [32]. Finally, because of skip pattern in the CIDI the questions on suicide attempt were only asked of those who reported ideation; while it is logical to assume that persons who have not seriously considered suicide would not have attempted it, this may have missed attempts that were more impulsive in nature. This study also has a number of strengths. The large, diverse, representative sample reduces the risk of selection bias, BED was assessed using a reliable, structured diagnostic instrument, and the analysis accounted for medical comorbidities that likely confound the BMI-suicidality relationship.

## Conclusions

Although more research is needed to decipher the complex relationships between BED, BMI, and suicidality, our findings can aid in understanding novel correlates of suicidality in the population. These findings complicate the ongoing debate about efforts to address eating disorders and the obesity endemic. With the adoption of BED in DSM-5, this will hopefully spur new conversations and debates about the relationships between weight, weight control messaging and interventions, and mental health.

## Additional files

Additional file 1: **Table S1.** Comparison of respondents excluded from main analysis due to missing data. **Table S2.** Relationship between binge eating and BMI and with suicidal behavior, excluding respondent with a history of BED. **Table S3.** Relationship between BMI and lifetime suicidal behavior, not accounting for binge eating behavior. **Table S4.** Relationship between BMI categories and lifetime suicidal behavior, not accounting for binge eating. (DOCX 30 kb)

Additional file 2: **Figure S1.** Probability of lifetime history of suicidality at select BMI. (DOCX 62 kb)

Additional file 3: **Figure S2.** Probability of past-year suicidality by lifetime binge eating behavior at select. (DOCX 24 kb)

## Abbreviations

BED: Binge Eating Disorder
BMI: Body mass index
CI: Confidence Interval
CIDI: Composite International Diagnostic Interview
CPES: Collaborative Psychiatric Epidemiologic Surveys
OR: Odds ratio
