# Supplementary material for: The relationship between body mass index, binge eating disorder and suicidality
Source: BMC Psychiatry. 2018 Jun 15;18:196. doi: 10.1186/s12888-018-1766-z (PMC6003111; doi:10.1186/s12888-018-1766-z)
Supplement: Supplementary file 1 — Table S1. Comparison of respondents excluded from main analysis due to missing data. Table S2. Relationship between binge eating and BMI and with suicidal behavior, excluding respondent with a history of BED. Table S3. Relationship between BMI and lifetime suicidal behavior, not accounting for binge eating behavior. Table S4. Relationship between BMI categories and lifetime suicidal behavior, not accounting for binge eating. (DOCX 30 kb) [file 12888_2018_1766_MOESM1_ESM.docx]

| **Table S1: Comparison of respondents excluded from main analysis due to missing data** | | |
| --- | --- | --- |
|  | **Included** | **Excluded due to missing data*** |
| N | 14,497 | 5,516 |
| Mean age (SE) | 42.9 (0.33) | 47.2 (0.58) |
| Female (N, %) | 8,528 (55.0) | 2,935 (47.1) |
| Race/ethnicity (N, %) |  |  |
| Asian | 2153 (6.6) | 131 (0.8) |
| Hispanic | 3128 (16.1) | 492 (5.0) |
| Black | 5501 (17.5) | 1021 (6.6) |
| Non-Hispanic White | 3715 (59.9) | 3872 (8.8) |
| More than high school education (N, %) | 7106 (49.6) | 2914 (53.3) |
| Income-to-needs ratio (N, %) |  |  |
| Quintile 1 | 4141 (24.0) | 495 (22.0) |
| Quintile 2 | 2522 (16.2) | 370 (16.3) |
| Quintile 3 | 3471 (25.3) | 534 (28.9) |
| Quintile 4 | 1825 (15.0) | 257 (14.4) |
| Quintile 5 | 2538 (19.6) | 270 (18.4) |
| Mean BMI (SE) | 27.5 (0.09) | 26.6 (0.10) |
| Lifetime history of suicidality (N, %) | 2205 (19.6) | 252 (6.1) |
| Lifetime history of suicide attempt (N, %) | 787 (7.8) | 44 (0.9) |
| Lifetime history of binge eating behavior (N, %) | 659 (3.9) | 31 (0.5) |
| Values are unweighted N, weighted percentage unless otherwise noted.  *Most missing observations occurred because only a subset of participants were assessed for BED in the NCSR. | | |

| Table S2: Relationship between binge eating and BMI and with suicidal behavior, excluding respondent with a history of BED | | | | | |
| --- | --- | --- | --- | --- | --- |
|  | **Model 1a**  **OR (95% CI), p-value** | **Model 1b**  **OR (95% CI), p-value** | **Model 2**  **OR (95% CI), p-value** | **Model 3**  **OR (95% CI), p-value** | **Model 4**  **OR (95% CI)** |
| Outcome: Lifetime suicidality |  |  |  |  |  |
| Lifetime binge eating | 1.92 (1.31 – 2.81), p<0.001 |  | 1.87 (1.28 – 2.72), p<0.001 | 1.79 (1.22 – 2.63), p<0.003 | 1.59 (1.01 – 2.51), p<0.047 |
| BMI (mean centered) |  | 1.00 (0.99 – 1.02), p<0.521 | 1.00 (0.99 – 1.02), p<0.571 | 1.01 (0.99 – 1.02), p<0.236 | 1.01 (0.99 – 1.03), p<0.090 |
| BMI^2^ |  | 1.00 (1.00 – 1.01), p<0.013 | 1.00 (1.00 – 1.01), p<0.014 | 1.00 (1.00 – 1.01), p<0.444 | 1.00 (1.00 – 1.01), p<0.446 |
| N | 14,226 | 14,226 | 14,226 | 14,226 | 9,462 |
| Outcome: Lifetime suicide attempt |  |  |  |  |  |
| Lifetime binge eating | 2.54 (1.45 – 4.44), p<0.001 |  | 2.46 (1.42 – 4.27), p<0.002 | 2.14 (1.21 – 3.78), p<0.009 | 1.76 (0.90 – 3.45), p<0.090 |
| BMI (mean centered) |  | 1.00 (0.98 – 1.02), p<0.768 | 0.99 (0.97 – 1.02), p<0.696 | 1.00 (0.98 – 1.03), p<0.873 | 1.01 (0.99 – 1.04), p<0.379 |
| BMI^2^ |  | 1.01 (1.00 – 101), p<0.004 | 1.00 (1.00 – 1.01), p<0.004 | 1.00 (1.00 – 1.01), p<0.216 | 1.00 (1.00 – 1.01), p<0.167 |
| N | 12,855 | 12,855 | 12,855 | 12,855 | 8,422 |
| Outcome: Past-year suicidality |  |  |  |  |  |
| Lifetime binge eating | 3.10 (1.53 – 6.26), p<0.002 |  | 3.07 (1.55 – 6.08), p<0.001 | 2.52 (1.27 – 4.98), p<0.008 | 2.37 (1.10 – 5.10), p<0.028 |
| BMI (mean centered) |  | 0.97 (0.95 – 1.02), p<0.411 | 0.98 (0.95 – 1.02), p<0.348 | 1.00 (0.97 – 1.04), p<0.873 | 1.01 (0.97 – 1.05), p<0.585 |
| BMI^2^ |  | 1.00 (1.00 – 1.01), p<0.001 | 1.00 (1.00 – 1.01), p<0.001 | 1.00 (1.00 – 1.01), p<0.204 | 1.00 (1.00 – 1.01), p<0.193 |
| N | 12,474 | 12,474 | 12,474 | 12,474 | 8,132 |
| Model 1a and 1b: Unadjusted. Model 2: Adjusted for BMI and BMI^2^. Model 3: Adjusted for BMI, race, gender, age, education, marital status, income to needs ratio, and number of chronic conditions. Model 4: Adjusted for BMI, race, gender, age, education, marital status, income to needs ratio, number of chronic conditions, and smoking status. | | | | | |

| Table S3: Relationship between BMI and lifetime suicidal behavior, not accounting for binge eating behavior | | | | |
| --- | --- | --- | --- | --- |
|  | **Model 1**  **OR (95% CI), p-value** | **Model 2**  **OR (95% CI), p-value** | **Model 3**  **OR (95% CI), p-value** | **Model 4**  **OR (95% CI), p-value** |
| Outcome: Lifetime suicidality |  |  |  |  |
| BMI (mean centered) | 1.01 (1.01 – 1.02), p<0.325 | 1.02 (1.01 – 1.04), p<0.005 | 1.01 (1.00 – 1.03), p<0.129 | 1.02 (1.00 – 1.03), p<0.046 |
| BMI^2^ | 1.00 (1.00 – 1.01), p<0.004 | 1.00 (1.00 – 1.00), p<0.238 | 1.00 (1.00 – 1.01), p<0.239 | 1.00 (1.00 – 1.00), p<0.253 |
| Chronic Count (ref. None) |  |  |  |  |
| One |  |  | 1.58 (1.36 – 1.83), p<0.001 | 1.46 (1.24 – 1.71), p<0.001 |
| Two |  |  | 2.49 (2.01 – 3.09), p<0.001 | 2.21 (1.75 – 2.81), p<0.001 |
| Three or more |  |  | 3.12 (2.53 – 3.84), p<0.001 | 2.67 (2.21 – 3.35), p<0.001 |
| Smoking status (ref. Never) |  |  |  |  |
| Former |  |  |  | 1.25 (0.99 – 1.59), p<0.063 |
| Current |  |  |  | 1.83 (1.58 – 2.12), p<0.001 |
| N | 14,497 | 14,497 | 14,497 | 9,648 |
| Outcome: Lifetime suicide attempt |  |  |  |  |
| BMI (mean centered) | 1.00 (0.98 – 1.02), p<0.985 | 1.02 (0.99 – 1.04), p<0.106 | 1.01 (0.98 – 1.03), p<0.619 | 1.01 (0.99 – 1.04), p<0.220 |
| BMI^2^ | 1.00 (1.00 – 1.01), p<0.003 | 1.00 (1.00 – 1.01), p<0.186 | 1.00 (1.00 – 1.01), p<0.153 | 1.00 (1.00 – 1.01), p<0.114 |
| Chronic Count (ref. None) |  |  |  |  |
| One |  |  | 2.07 (1.62 – 2.65), p<0.001 | 1.87 (1.44 – 2.44), p<0.001 |
| Two |  |  | 3.59 (2.75 – 4.69), p<0.001 | 3.03 (2.31 – 3.96), p<0.001 |
| Three or more |  |  | 4.98 (3.45 – 7.18), p<0.001 | 3.86 (2.64 – 5.66), p<0.001 |
| Smoking status (ref. Never) |  |  |  |  |
| Former |  |  |  | 1.85 (1.38 – 2.48), p<0.001 |
| Current |  |  |  | 3.36 (2.58 – 4.39), p<0.001 |
| N | 13,079 | 13,079 | 13,079 | 8,577 |
| Model 1: Unadjusted. Model 2: Adjusted for race, gender, age, education, marital status, and poverty-to-income ratio. Model 3: Adjusted for race, gender, age, education, marital status, poverty-to-income ratio, and number of chronic conditions. Model 4: Adjusted for race, gender, age, education, marital status, poverty-to-income ratio, number of chronic conditions, and smoking status. | | | | |

| Table S4: Relationship between BMI categories and lifetime suicidal behavior, not accounting for binge eating | | | | |
| --- | --- | --- | --- | --- |
|  | **Model 1**  **OR (95% CI)** | **Model 2**  **OR (95% CI)** | **Model 3**  **OR (95% CI)** | **Model 4**  **OR (95% CI)** |
| Outcome: Lifetime suicidality |  |  |  |  |
| BMI group (ref. 25 to <30) |  |  |  |  |
| <25kg/m^2^ | 1.02 (0.90 – 1.16) | 0.87 (0.75 – 1.00) | 0.88 (0.76 – 1.03) | 0.84 (0.71 – 0.99) |
| 25 to <30kg/m^2^ | 1.0 | 1.0 | 1.0 | 1.0 |
| 30 to <35 kg/m^2^ | 1.04 (0.87 – 1.23) | 1.03 (0.86 – 1.22) | 0.95 (0.80 – 1.13) | 0.95 (0.78 – 1.16) |
| ≥35 kg/m^2^ | 1.49 (1.21– 1.84) | 1.36 (1.09 – 1.70) | 1.17 (0.92 – 1.50) | 1.22 (0.91 – 1.63) |
| N | 14,497 | 14,497 | 14,497 | 9,648 |
| Outcome: Lifetime suicide attempt |  |  |  |  |
| BMI group (ref. 25 to <30) |  |  |  |  |
| <25kg/m^2^ | 1.11 (0.88 – 1.42) | 0.90 (0.70 – 1.15) | 0.92 (0.72 – 1.17) | 0.85 (0.65 – 1.12) |
| 25 to <30kg/m^2^ | 1.0 | 1.0 | 1.0 | 1.0 |
| 30 to <35 kg/m^2^ | 1.01 (0.77 – 1.32) | 0.98 (0.76 – 1.26) | 0.88 (0.68 – 1.13) | 0.86 (0.64 – 1.16) |
| ≥35 kg/m^2^ | 1.77 (1.20 – 2.61) | 1.50 (1.00 – 2.24) | 1.23 (0.80 – 1.89) | 1.42 (0.88 – 2.30) |
| N | 13,079 | 13,079 | 13,079 | 8,577 |
| Model 1: Unadjusted. Model 2: Adjusted for race, gender, age, education, marital status, poverty-to-income ratio. Model 3: Model 2 plus additionally adjusted for number of chronic conditions. Model 4: Model 3 plus additionally adjusted for smoking | | | | |
