# Supplementary material for: The relationship between body mass index, binge eating disorder and suicidality
Source: BMC Psychiatry. 2018 Jun 15;18:196. doi: 10.1186/s12888-018-1766-z (PMC6003111; doi:10.1186/s12888-018-1766-z)
Supplement: Supplementary file 2 — Figure S1. Probability of lifetime history of suicidality at select BMI. (DOCX 62 kb) [file 12888_2018_1766_MOESM2_ESM.docx]

**Figure S1: Probability of lifetime history of suicidality at select BMI**

Marginal probability of suicidality

BMI (kg/m^2^)

Figure S1 Caption: Marginal predicted probability of lifetime history of suicidal ideation/attempt at select values of BMI. Values are estimated at the sample mean for all model covariates (age, gender, race/ethnicity, marital status, income-to-needs ratio and chronic conditions).
