# Supplementary material for: The relationship between body mass index, binge eating disorder and suicidality
Source: BMC Psychiatry. 2018 Jun 15;18:196. doi: 10.1186/s12888-018-1766-z (PMC6003111; doi:10.1186/s12888-018-1766-z)
Supplement: Supplementary file 3 — Figure S2. Probability of past-year suicidality by lifetime binge eating behavior at select. (DOCX 24 kb) [file 12888_2018_1766_MOESM3_ESM.docx]

**Figure S2: Probability of past-year suicidality by lifetime binge eating behavior at select BMI**

BMI (kg/m^2^)

Marginal probability of suicidality

Figure S2 Caption: Marginal predicted probability of past year suicidal ideation/attempt by lifetime history of binge eating behavior at select values of BMI. Values are estimated at the sample mean for all model covariates (age, gender, race/ethnicity, marital status, income-to-needs ratio and chronic conditions). N=14,497.
